# Richer gut microbiota with distinct metabolic profile in HIV infected Elite Controllers

**DOI:** 10.1038/s41598-017-06675-1

**Published:** 2017-07-24

**Authors:** Jan Vesterbacka, Javier Rivera, Kajsa Noyan, Mariona Parera, Ujjwal Neogi, Malu Calle, Roger Paredes, Anders Sönnerborg, Marc Noguera-Julian, Piotr Nowak

**Affiliations:** 1 Department of Medicine Huddinge, Unit of Infectious Diseases, Karolinska Institutet, Karolinska University Hospital, Stockholm, Sweden; 2 IrsiCaixa & AIDS Unit, Hospital Universitari Germans Trias i Pujol, Universitat Autònoma de Barcelona, Badalona, Spain; 3 Department of Laboratory Medicine, Division of Clinical Microbiology, Karolinska Institutet, Karolinska University Hospital, Stockholm, Sweden; 4 Universitat de Vic-Universitat Central de Catalunya, Catalonia, Spain; 5 Universitat Autònoma de Barcelona, 08193 Bellaterra Catalonia, Spain; 6 HIV Unit & Lluita Contra la SIDA Foundation, Hospital Universitari Germans Trias i Pujol, Ctra de Canyet s/n, 08916 Badalona, Catalonia Spain

## Abstract

Gut microbiota dysbiosis features progressive HIV infection and is a potential target for intervention. Herein, we explored the microbiome of 16 elite controllers (EC), 32 antiretroviral therapy naive progressors and 16 HIV negative controls. We found that the number of observed genera and richness indices in fecal microbiota were significantly higher in EC versus naive. Genera *Succinivibrio, Sutterella, Rhizobium, Delftia, Anaerofilum* and *Oscillospira* were more abundant in EC, whereas *Blautia* and *Anaerostipes* were depleted. Additionally, carbohydrate metabolism and secondary bile acid synthesis pathway related genes were less represented in EC. Conversely, fatty acid metabolism, PPAR-signalling and lipid biosynthesis proteins pathways were enriched in EC vs naive. The kynurenine pathway of tryptophan metabolism was altered during progressive HIV infection, and inversely associated with microbiota richness. In conclusion, EC have richer gut microbiota than untreated HIV patients, with unique bacterial signatures and a distinct metabolic profile which may contribute to control of HIV.

## Introduction

Progressive HIV-1 infection is characterized by depletion of CD4+ T cells in gut-associated lymphoid tissue, followed by immune activation, gut microbiota dysbiosis, and microbial translocation^1–3^. Elite controllers (EC) constitute less than 1% of the HIV-infected population^4^, and have sustained viral suppression in absence of antiretroviral therapy (ART). Due to definitional bias, a high rate of heterogeneity is observed among EC cohorts^5^. It appears that host genetic rather than demographic factors contribute to the viral controlling properties, e.g. with an increased rate of HLA B*5701 allele positivity. Also, unique immunological cellular responses against HIV-1 have been proposed as a mechanism for viral control^6^. Despite spontaneous suppressed plasma viremia, microbial translocation and immune activation are present in EC^7^.

The gut microbial composition in EC has not been extensively explored, with only three studies (with low numbers of subjects) investigating their gut microbiome^8–11^. In a previous work, differences in the bacterial composition of gut microbiota between ART naive HIV patients and EC were observed at the phylum level, with an enrichment of Bacteroidetes and a reduction of Actinobacteria in EC^9^. It was also found that the EC had lower beta-diversity (i.e. inter-individual variation in the gut microbiota) than the viremic patients, and principal coordinate analysis (PCoA) revealed that EC clustered separately, indicating a different gut microbiome compared to other HIV-infected individuals.

Use of metagenomic techniques has illuminated the complex interactions between the host metabolic activities and gut microbial species in several diseases^12^. Thus, alterations in the catabolism of tryptophan have been linked to progressive HIV-infection, and correlated with a pathological shift in the gut microbiota^10^. In depth, tryptophan degradation products have been linked to loss of Th17/regulatory T cell balance fueling the chronic inflammation in progressive HIV disease^13^. Whether the gut microbiota in EC differently influences the tryptophan metabolism has not been explored, but markers of tryptophan catabolism were not elevated in EC as compared to healthy subjects^11^.

In the current work, we investigated if HIV infection differently affects the gut microbiome in patients with progressive HIV infection and EC. We also explored the link between the composition, inferred functionality of gut microbiome and systemic inflammatory, immunological and metabolic markers in these patients.

## Material and Methods

### Study design

This was a cross-sectional study including both HIV seropositive and seronegative participants.

### Patients

Detailed characteristics are presented in Table 1. Totally, 48 study subjects were recruited from the out-patient HIV clinic at Karolinska University Hospital, Stockholm, Sweden. Additionally we included 16 HIV negative controls (negative). Inclusion criteria were age >18 years, HIV positive for at least 6 months and no ongoing HIV-related complications. All viremic progressors had to be ART naive (naive). Exclusion criteria were inflammatory bowel disease or infectious gastroenteritis within the last four weeks. EC were defined by: (I) HIV positive for ≥1 year and with ≥3 consecutive viral loads (VLs) <75 c/ml over one year with all previous VLs < 1000 c/m, or (II) HIV positive for ≥10 years, with ≥2 VLs and ≥90% of all VLs < 400 c/ml. Four female EC had been on short time ART due to pregnancy (three for 3.5 months, one for 14 days), all more than four years before study entry. The study subjects were categorized into three groups (EC: n = 16; naive: n = 32; negative: n = 16) and were matched by Body Mass Index (BMI), age, gender and sexual practice. All participants gave written informed consent. All the work and experiments were performed in accordance with relevant guidelines, regulations and with the Declaration of Helsinki. The study was approved by the Regional Ethics Committee at Karolinska University Hospital, Stockholm (2009/1485-31, 2013/1944-31/4, 2014/920-3).

**Table 1:** Cohort demographics and cellular immune activation markers at baseline.

	EC	Naive	Negative	p-value
Number of individuals	16	32	16	
Age (years, median (IQR))^*^	47 (40.3–54.3)	43.5 (37.3–50.5)	49 (44–52.8)	ns
Gender (n, male/female)^†^	9/7	16/16	8/8	ns
**Ethnicity (n)**				
Black	9	13	0	
Caucasian	6	17	15	
Latin	1	1	0	
Oriental	0	1	1	
**Mode of transmission (n)**				
Heterosexually	8	21	NA	
MSM	4	8		
IVDU	1	3		
Blood transfusion	2	0		
Unknown	1	0		
**Sexual practice** ^**†**^				
Heterosex	12	24	12	ns
MSM	4	8	4	
Time since diagnosis (years, median (IQR))^*^	8.55 (5.0–18.0)	3 (0.7–6.9)	NA	0.0008
Body Mass Index (BMI) (score (IQR))^*^	26.4 (24.1–32.2)	25 (23.0–30.0)	24.2 (22.9–25.6)	ns
CD4+ T-cell count (median (IQR)^*^	806 (676–1049)	390 (298–475)	NA	<0.0001
CD8+ T-cell count (median (IQR)*	705 (541–904)	995 (678–1373)	NA	0.02
CD4/CD8+ T-cell ratio (median (IQR)^*^	1.41 (0.74–1.55)	0.38 (0.27–0.51)	NA	<0.0001
CD4+ T-reg cells (FoxP3+CD25+) % (median (IQR)*	4.91 (4.13–5.66)	NA	5.88 (4.77–7.28)	ns
CD4+ HLA-DR+ CD38+ T cells % (median (IQR))*	0.44 (0.34–0.75)	7.78 (5.34–14.8)	0.53 (0.39–0.61)	<0.0001
CD8+ HLA-DR+ CD38+ T cells % (median (IQR))*	1.16 (0.77–1.6)	36.9 (23.6–44.9)	0.71 (0.52–1.71)	<0.0001

EC = elite controllers. Naive = viral progressors. Negative = negative controls.

*Kruskal-Wallis test was used for comparison between three groups, and Dunn’s Multiple Comparison Test was adapted for “post hoc” testing. Mann-Whitney was applied for comparisons between two groups. ^†^Chi-square test was applied. NA (not available). ns (non significant) indicates p-value > 0.05.

### Blood Sample Collection and Isolation of Peripheral Blood Mononuclear Cells

Plasma, isolated from EDTA-treated peripheral blood, and serum samples were stored at −80 °C until analyses. Peripheral blood mononuclear cells (PBMCs) were isolated from EDTA-treated blood using Hypaque-Ficoll (GE Healthcare) density gradient centrifugation, counted with Nucleocounter® and then cryopreserved at −150 °C in fetal bovine serum (Sigma-Aldrich) containing 10% DMSO (Sigma-Aldrich), at a concentration of 10^6^ cells/ml of cryopreservation media. Soluble markers of inflammation and microbial translocation, and metabolites of tryptophan catabolism pathway were analyzed in plasma by ELISA (hs-CRP (Abcam, UK), sCD14 (R&D, Minnesota, USA), IL-6 (R&D), LBP (Hycult Biotech, The Netherlands)) or HPLC (http://bevital.no), respectively, according to manufacturer’s instructions.

### Flow Cytometry, Immunophenotyping, and Viral Load

Quantification of CD4+ and CD8+ T-cells and plasma HIV-1 RNA were performed as part of the clinical routine with flow cytometry and Cobas Amplicor (Roche Molecular Systems Inc., Branchburg, New Jersey, USA), respectively. At the day of analysis, cryopreserved PBMCs were thawed and stained for HLA-DR and CD38 as markers of immune activation of CD4+ and CD8+ T- cells, and FoxP3 and CD25 as markers of CD4+ T-regulatory cells^14^. HIV negative samples were not analyzed by routine flow cytometry, which is mirrored by the lack of CD4+ and CD8+ T-cell total counts in that group (Table 1).

### Fecal Sample Collection

A sterile tube for fecal sampling without preservation media was used when participants were able to donate feces adjacent to their study visit at the clinic. The sample was frozen and stored at −80 °C within 24 hours. PSP^®^ Spin Stool DNA sampling tube (Stratec Biomedical) was used for participants who submitted feces at home. The stool samples were delivered to the out-patient clinic by the participant, or instantly sent by post and stored at −70 °C according to the manufacturer’s instructions^15^. All participants were asked to complete a standardized questionnaire, collecting data about recent use of antibiotics (last 3 months) and probiotics, current medication, alcohol use, smoking, chronic diseases, recent infectious gastroenteritis (last 4 weeks), special diet (vegan/vegetarian/gluten-/lactose- free), colectomy, recent travelling abroad (>4 weeks last 12 months) and time since arrival in Sweden for non-natives.

### DNA extraction, 16s rRNA gene amplification and Sequencing

DNA extraction was performed using the PowerSoil DNA Extraction Kit (MO BIO Laboratories, Carlsbad, CA, US). To amplify the variable region V3-V4 from the 16S rRNA gene (amplicon size expected ~460 bp), we used the primer pair described in the MiSeq rRNA Amplicon Sequencing protocol which already have the Illumina adapter overhang nucleotide sequences added to the 16S rRNA V3-V4 specific-primers, i.e.: 16S_F 5′-(TCG TCG GCA GCG TCA GAT GTG TAT AAG AGA CAG **CCT ACG GGN GGC WGC AG**)-3′ and 16S_R 5′-(GTC TCG TGG GCT CGG AGA TGT GTA TAA GAG ACA G**GA CTA CHV GGG TAT CTA ATC C**)-3′.

Amplifications were performed in triplicate 25 μL reactions, each containing 2.5 μL of non-diluted DNA template, 12.5 μL of KAPA HiFi HotStart Ready Mix (containing KAPA HiFi HotStart DNA Polymerase, buffer, MgCl_2_, and dNTPs, KAPA Biosystems Inc., Wilmington, MA, USA), and 5 μL of each primer at 1 μM. Thermal cycling conditions consisted of an initial denaturation step (3 min at 95 °C), followed by 30 cycles of denaturation (30 sec at 95 °C), annealing (30 sec at 55 °C) and extension (30 sec at 72 °C). These were followed by a final extension step of 10 min at 72 °C. Once the desired amplicon was confirmed in 1% agarose gel electrophoresis, all three replicates were pooled and stored at −30 °C until sequencing library preparation. Amplified DNA templates were cleaned-up for non-DNA molecules and Illumina sequencing adapters and dual indices were attached using Nextera XT Index Kit (Illumina, Inc.) followed by the corresponding PCR amplification program as described in the MiSeq 16S rRNA Amplicon Sequencing protocol. After a second round of cleanup, amplicons were quantified using Quant-iT™ PicoGreen® dsDNA Assay Kit (Invitrogen, Carlsbad, MA, USA) and diluted in equimolar concentrations (4 nM) for further pooling. Sequencing was performed on an Illumina MiSeq^TM^ platform according to the manufacturer’s specifications to generate paired-end reads of 300 base-length in each direction.

### Data Analysis

Sequencing data was processed using Mothur^16^ phylotype approach. Briefly, paired-end data were merged and quality filtered and all reads not matching the used V3-V4 amplicon design were discarded. Chimeric sequences were filtered using Mothur Uchime^17^ implementation. Sequences were classified using RDP algorithm^18^ in combination with 16s rRNA Silva database^19^. Obtained sequences from five subjects (one EC, three naive and one negative) were of poor quality and were excluded from further analyses. To assess alpha diversity, richness (Chao1 and ACE) and diversity (Shannon and Simpson) indices were computed using R/vegan library^20, 21^ selecting a subsample of ten thousand counts for each individual.

Bacterial genera count table were normalized to relative abundance measures. These were used to compute Bray – Curtis^22^ dissimilarity between each pair of individuals, which was used as input ordination analysis using non-metric multidimensional scaling (NMDS). Correlation between NMDS plot axis coordinates and inflammation parameters were tested by applying Spearman test. Additionally, a PERMANOVA (adonis) test was performed on this distance matrix to partition different sources of variation using R/vegan package.

Microbiome function was inferred using PICRUSt^23^ on GreenGenesDB^24^ classified phylotypes. Counts were normalized by considering 16s rRNA gene copy number. To infer the gene content, the normalized phylotype abundances were multiplied by the respective set of gene abundances, represented by Kyoto Encyclopedia of Genes and Genomes (KEGG) identifiers estimated for each taxon.

### Statistics

Multiple group differences in diversity indices, inflammation and activation markers and bacterial abundances were analyzed via Kruskal–Wallis rank-based test. Benjamini–Hochberg^25^ correction was used to correct for multiple testing. Two-tailed Mann-Whitney U-test was applied for comparisons of inflammation markers between two groups.

Inflammation indices were associated both with genus and functional composition using Spearman correlation. Associations with a Benjamini–Hochberg adjusted p-value lower than 0.01 were considered as relevant and inflammation parameters associated with less than two bacteria were discarded when plotting the heatmap. Bacterial genus and functions were ordered in the heatmap according to a clustering between them using Ward hierarchical clustering.

With the aim of evaluating the power of the classification of individuals according to their microbiome composition profile, a LASSO penalized logistic regression model as proposed in the bibliography^26^ was computed for each pair of profiles. LiblineaR and pROC libraries were used to obtain the regression models, represent ROC curves and estimate model accuracy using AUC.

### Data Availability

Metagenomics raw sequencing data along with sample level metadata have been deposited using the NCBI/SRA Web service and compliance to MIMARKS standard. Data can be accessed using BioProject accession number PRJNA354863.

## Results

This was a cross-sectional study including 64 participants (Table 1). The groups were balanced by age, gender, sexual practice and BMI. The heterosexual transmission route was slightly more common in the naive (65.6 vs 50.0%), whilst the rate of the MSM transmission route was the same in both groups. Two naive patients had chronic hepatitis B infection, whereas two EC and two naive had chronic hepatitis C infection. Use of antibiotics within three months before inclusion was declared from 2 EC, 6 naive and 2 negative. One EC was vegetarian, one EC and one negative were on lactose/gluten-free diet (Supplementary Table 1). The median viral load (copies/mL) of EC was <20 (75% percentile 30.25), of naive 31700 (IQR 4430-100250).

### Comparable T-cell activation in Elite Controllers and Negative

As expected, BL CD4+ T-cell count was lower and CD8+ T-cell count significantly higher in naive vs EC. Proportions of CD4+ T-regulatory cells tended to be higher in negative compared to EC (p = 0.07). The level of immune activation of CD4+ and CD8+ T-cells in blood (CD4/8+ T-cell ratio and by expression of HLA-DR+ CD38+) was similar in EC and negative but significantly lower compared to naive group (Table 1).

### Richness, diversity and composition of fecal microbiota

**Figure 1:**
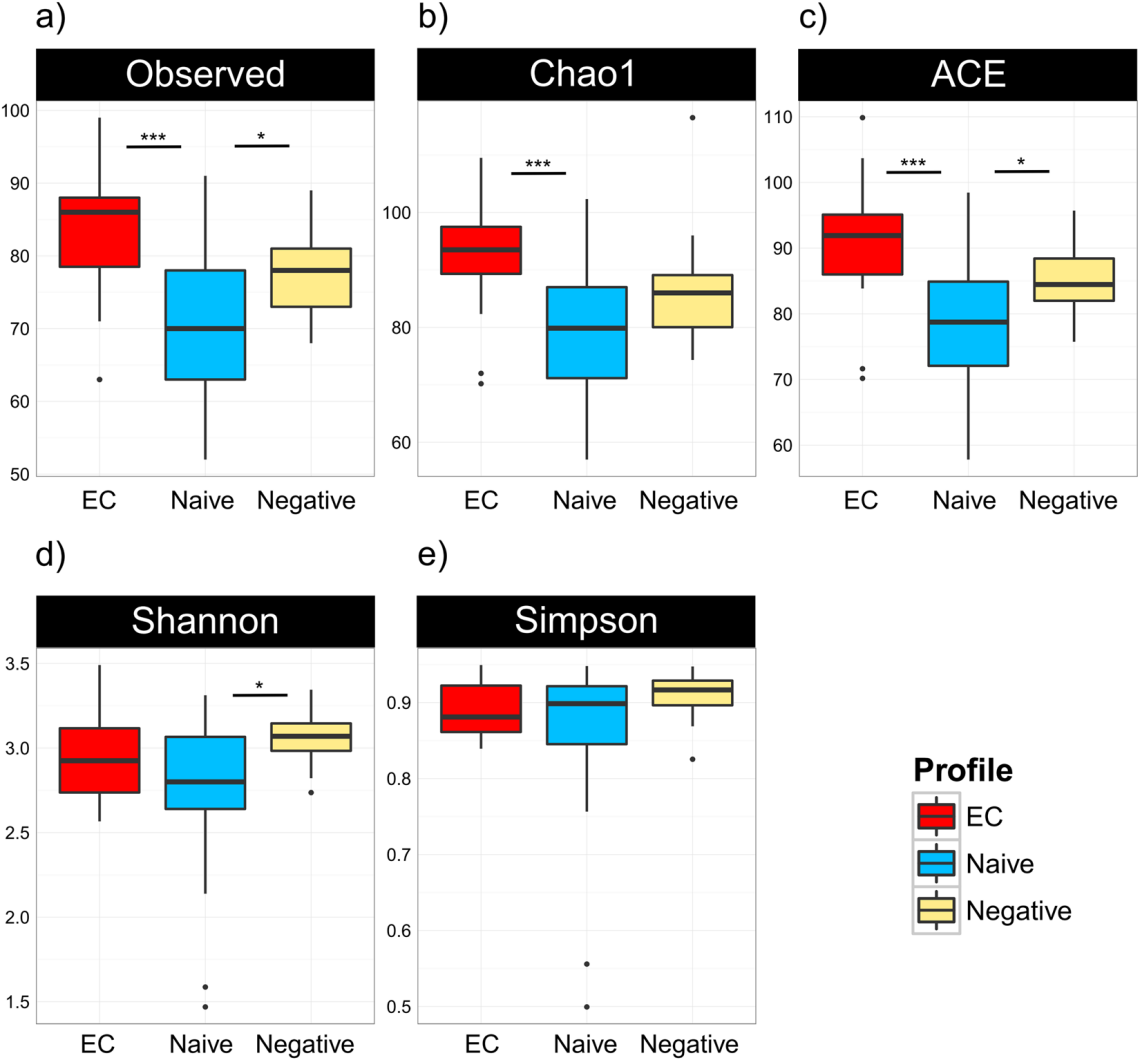
Similar richness and diversity of fecal microbiota in EC and negative controls. Number of observed bacterial genera was significantly lower in naive patients as compared to the other groups (**a**). Richness indices Chao-1 (**b**) and ACE (**c**) were reduced in naive, but no significant differences were observed between EC and negative. Alpha-diversity, assessed by Shannon index was lower in naive as compared to negative (**d**), whereas Simpson index was similar in all groups (**e**). Comparisons between groups were obtained via Kruskal-Wallis rank based test including Dunn’s post-hoc pairwise analyses. Benjamini-Hochberg method was used for correction of multiple testing. A p-value < 0.05 was considered significant. Box plots represent median (black horizontal line), 25th and 75th quartiles (edge of boxes), upper and lower extremes (whiskers). Outliers are represented by a single data point.

**Figure 2:**
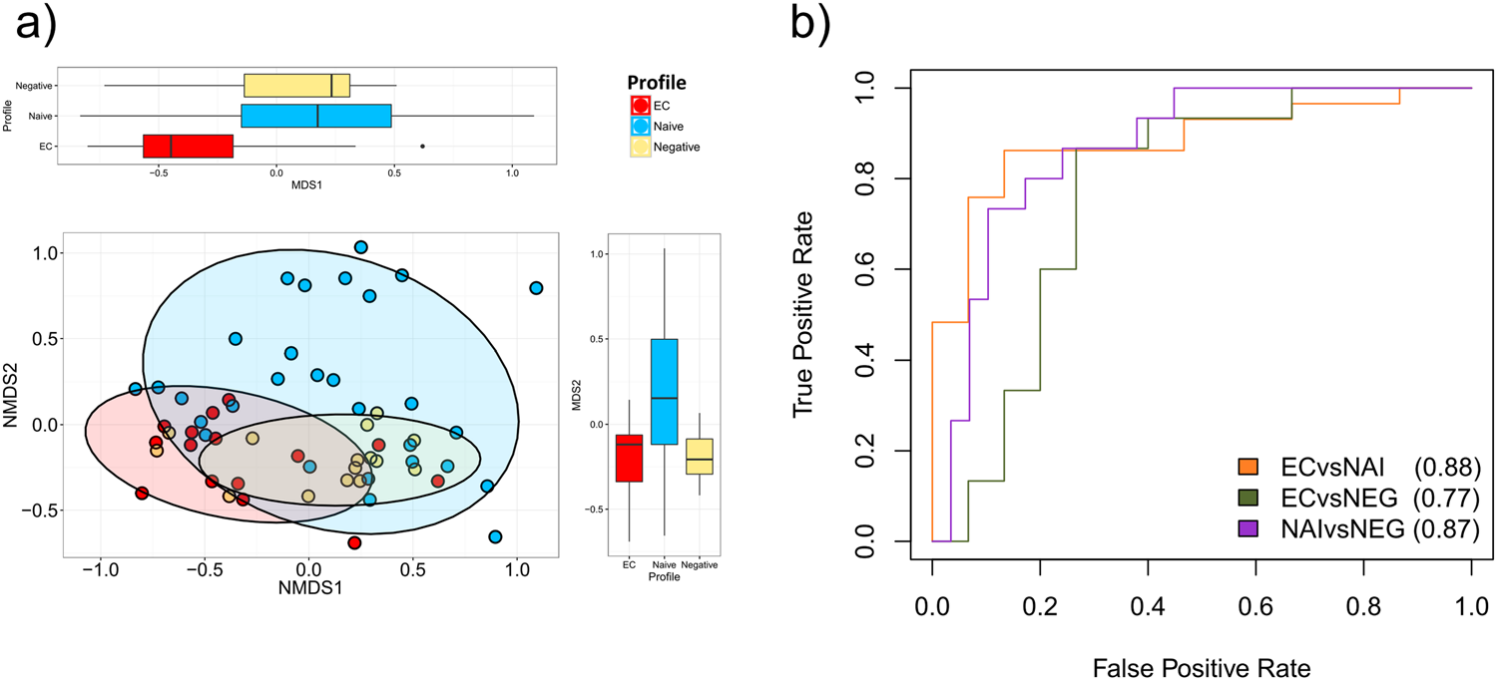
Separation between EC and naive patients in inter-individual (ß-diversity) analyses. Non-metric multidimensional scaling (NMDS) analysis was performed to characterize inter-individual differences between groups, revealing clustering of EC at NMDS axis 1 and naive at axis 2. The separations between groups at each axis are presented in respective box-plot. Box plots represent median (black horizontal line), 25th and 75th quartiles (edge of boxes), upper and lower extremes (whiskers). Outliers are represented by a single data point (**a**). LASSO regression analysis with AUROC (ROC curves; AUC used for estimation of model accuracy) curve was used for classification of gut microbiota composition between groups, and lowest accuracy was found between EC and negative patients (AUC 0.77, suggesting that the similarity of microbiota composition was highest between these groups) (**b**).

**Figure 3:**
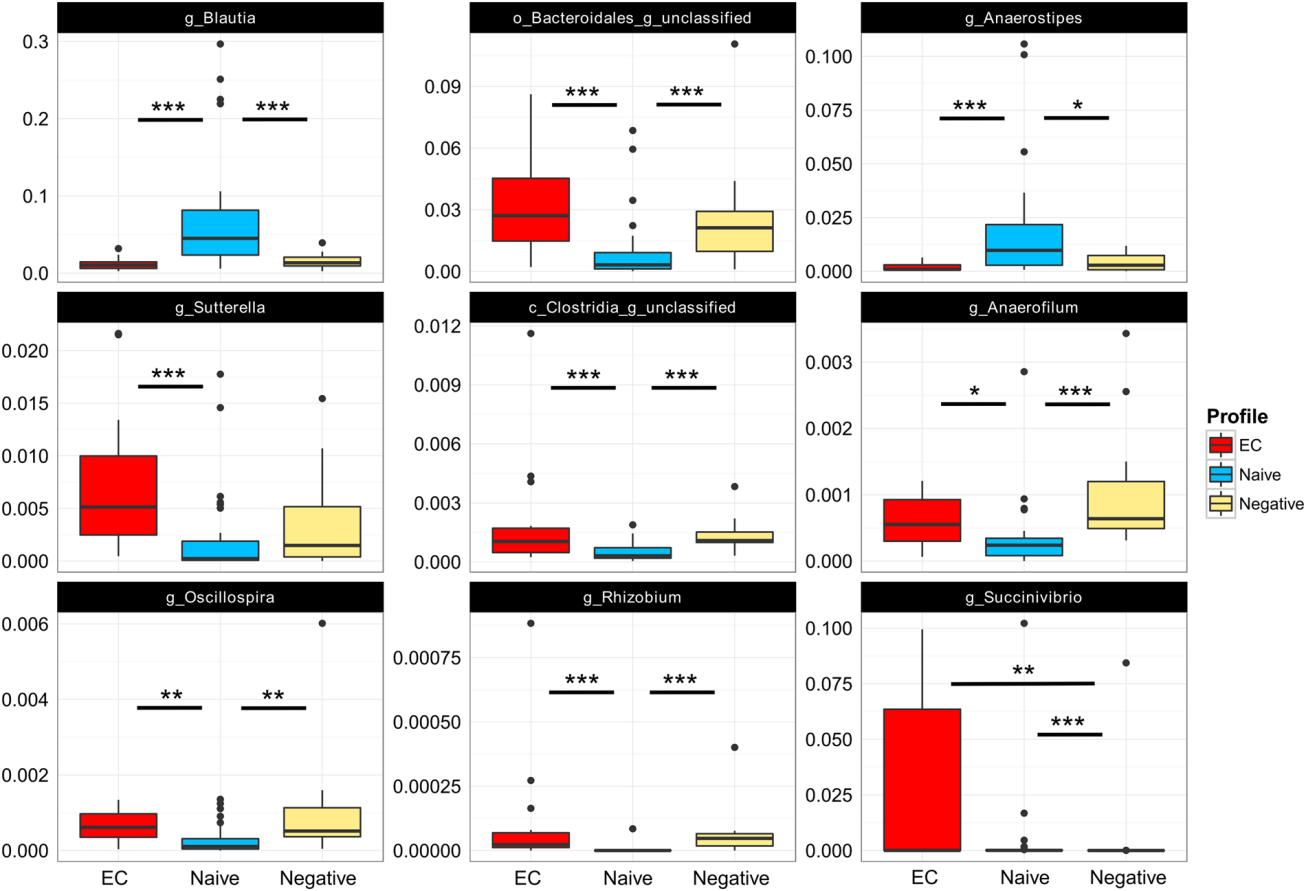
Compositional differences in fecal microbiota between groups. Several differences in bacterial abundance were observed between the groups at genus level. Comparisons of taxa abundances were performed via Kruskal -Wallis rank based test and Benjamini-Hochberg method was used for correction of multiple testing. Adjusted p-value < 0.01 was considered significant for Kruskal-Wallis. Dunn’s post-hoc pairwise analyses: *p < 0.05, **p < 0.01, ***p < 0.001. Box plots represent median (black horizontal line), 25th and 75th quartiles (edge of boxes), upper and lower extremes (whiskers). Outliers are represented by a single data point.

Overall, the fecal microbiota was richer and more diverse in EC as compared to naives and similar to negative. Thus, the number of observed taxa in fecal microbiota was higher in EC vs naive (Δ 19.8; p = 0.0001), and not different compared to negative (Δ 8.3; p = 0.14) (Fig. 1a). Similarly, naive patients had decreased estimated richness indices Chao 1 (EC-naive: Δ 19.6; p = 0.0002, EC-negative: Δ 10.4; p = 0.07, naive-negative Δ −9.2; p = 0.007) and ACE (EC-naive: Δ 20.5; p = 0.0001, EC-negative: Δ 9.7; p = 0.09, naive-negative Δ − 10.8; p = 0.03); (Fig. 1b,c). The Shannon index was increased in negative group as compared to naive (Δ − 13.5; p = 0.01) (Fig. 1d) suggesting HIV induced changes in alpha diversity in the latter group. To further characterize the inter-individual differences between groups (beta-diversity) at group level, non-metric multidimensional scaling (NMDS) and LASSO regression analysis with ROC curve and AUC were performed. NMDS analysis revealed separation and clustering of EC along NMDS1 axis, whilst naive tended to cluster along NMDS2 (Fig. 2a). The lowest accuracy of LASSO regression was found when using microbiome composition to classify EC vs negative patients (AUC = 0.77), confirming that the gut microbiota composition was least different among these individuals. Additionally, LASSO classification was more accurate when classifying naive vs either EC (AUC = 0.88) and negative (0.87) (Fig. 2b). Furthermore, PERMANOVA (adonis) test yielded that the bacterial composition varied between the groups (R^2^ = 0.12; p = 0.001). The groups differed significantly in abundance of 17 bacterial taxa at the genus level (Fig. 2 and Supplementary material Figure S1). We found that genera of *Succinivibrio* and *Sutterella* were enriched in EC only. Additionally, *Rhizobium, Delftia, Anaerofilum* and *Oscillospira* genera were more abundant in EC than in naive, but not significantly different from negative. Moreover, genus *Blautia* and *Anaerostipes* were enriched in naive as compared to EC and negative (Fig. 3). We also found significant differences in abundance of unclassified genera at higher taxonomic levels between the groups (Supplementary material Figure S1).

### Inferred gut microbiota functionality

**Figure 4:**
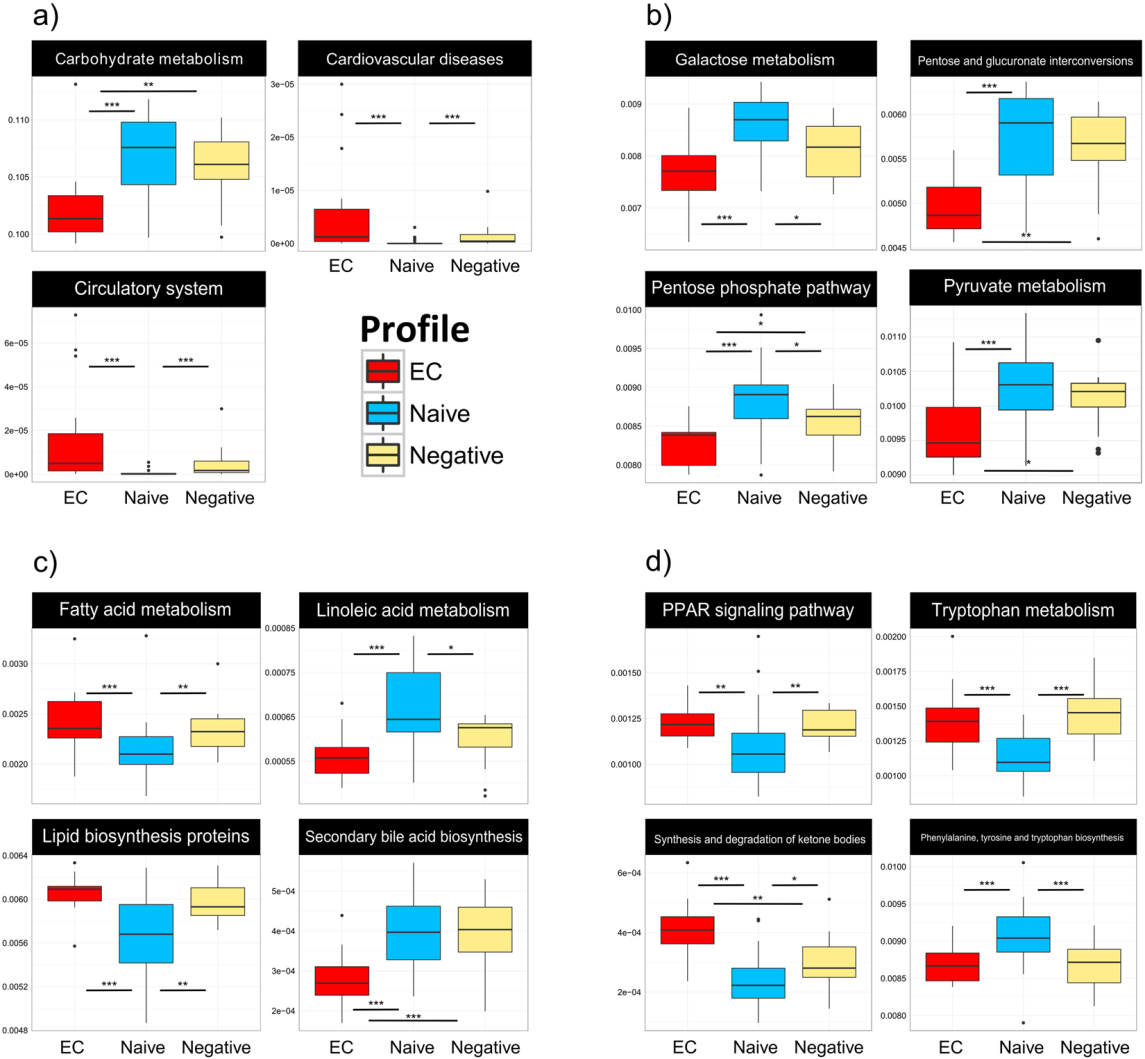
Inferred functional content of gut microbiota. The metagenomic functional content of gut microbiota was predicted by inferred PICRUSt analysis. Abundance of pathways involved in carbohydrate metabolism, cardiovascular diseases and circulatory system at KEGG level II (**a**), or level III (**b**–**d**). Pathways involved in carbohydrate metabolism, galactose metabolism, pentose and glucoranate interconversions, pentose-phosphate pathway and pyrovate metabolism (**b**). Pathways related to metabolism of lipids and fatty acids and biosynthesis of secondary bile acids (**c**). Bacterial tryptophan metabolism, PPAR signaling, phenylalanine, tyrosine and tryptophan biosynthesis and synthesis and degradation of ketone bodies pathways (**d**). Kruskal – Wallis rank-based test was applied, and Benjamini – Hochberg method was used to correct for multiple testing. Adjusted p-value < 0.01 was considered significant for Kruskal-Wallis. Dunn’s post-hoc pairwaise analyses: *p < 0.05, **p < 0.01, ***p < 0.001. Box plots represent median (black horizontal line), 25th and 75th quartiles (edge of boxes), upper and lower extremes (whiskers). Outliers are represented by a single data point.

The PICRUSt analysis, predicting the metagenomic functional content of gut microbiota, revealed several significant differences between the groups at both KEGG level II and III. Hence, at the KEGG level II, we found that the predicted pathway of carbohydrate metabolism was significantly reduced in the gut bacterial metagenome of EC as compared to both naive and negative patients. Instead, genes encoding cardiovascular diseases and circulatory system pathways were enriched in EC as compared to naive, but were not significantly different as compared to negative (Fig. 4a). Moreover, several pathways related to the metabolism of carbohydrates were decreased in EC in relation to naive and negative at KEGG level III. Thus, galactose metabolism, pentose-glucoronate interconversions, pyruvate metabolism and pentose-phosphate pathway (PPP) were predicted to have a lower abundance in EC vs naive. PPP was significantly reduced in EC as compared to all other groups, and both galactose and PPP were significantly more abundant in naive vs negative (Fig. 4b). Pathways related to lipid metabolism were differentially distributed in the metagenome of the cohort. Those involved in metabolism of fatty acids and lipid biosynthesis proteins were significantly reduced in naive as compared to the other groups. Conversely, the essential fatty acid linoleic acid metabolism pathway was more represented in naive. The metagenomic proportion of secondary bile acid biosynthesis metabolism pathway, which has a key function in cholesterol homeostasis, was significantly reduced in EC, but present at similar level in naive and negative (Fig. 4c). We also found that the PPAR (peroxisome proliferator-activated receptors)-signaling pathway, which plays an essential role in metabolism of carbohydrates, lipids and proteins, was significantly reduced in naive. Additionally, pathways related to synthesis and degradation of ketone bodies were reduced in naive, whereas significantly enriched in EC, also when compared to negative. Tryptophan metabolism related genes were decreased in naive vs negative. In contrast, proportions of phenylalanine, tyrosine and tryptophan biosynthesis pathway were enriched in naive (Fig. 4d). Additional functional pathways with different distribution in the cohort are presented in supplementary material (Figure S2).

### Plasma levels of soluble markers of inflammation and tryptophan catabolism metabolites

Plasma levels of soluble markers of inflammation, immune activation and metabolites related to the kynurenine pathway of tryptophan degradation are presented in Table 2. We found that EC had higher levels of IL-6 and hs-CRP than negative; however levels of soluble immune activation marker sCD14 were not different among groups. Levels of LBP, commonly used as a marker of microbial translocation, were significantly increased in naive group as compared to others.

**Table 2:** Soluble markers of inflammation and metabolites of kynurenine/tryptophan catabolism in plasma.

	EC	Naive	Negative	p-value*
**Soluble marker: median(IQR)**				
LBP (ng/ml)	3727 (2206–15123)	6805 (5984–8031)	2862 (1956–3705)	0.0004
sCD14 (pg/ml)	1.7 × 10^6^ (1.53–1.95 × 10^6^)	1.47 × 10^6^ (1.46–1.71 × 10^6^)	1.5 × 10^6^ (1.44–1.65 × 10^6^)	ns
IL-6 (pg/ml)	1.73 (1.18–3.20)	NA	0.84 (0.67–1.78)	0.035
hs-CRP (pg/ml)	1.37 × 10^6^ (0.76–2.7 × 10^6^)	NA	635419 (378718–941463)	0.005
**Tryptophan catabolism:**				
Tryptophan (umol/L)	53.1 (51.4–60.5)	46.2 (40.5–50.8)	66.1 (60.3–73.1)	<0.0001
Kynurenine (umol/L)	1.4 (1.3–1.6)	1.65 (1.3–2)	1.7 (1.4–1.9)	ns
Anthralinic acid (nmol/L)	12.2 (10.1–16.2)	21.6 (16.3–28.8)	15.3 (11.6–20.6)	0.0007
Kynurenic acid (nmol/L)	40.5 (36.2–50.6)	30.3 (17.6–48.9)	54.2 (48.9–70.2)	0.0015
3-Hydroxykunrenin (nmol/L)	45.9 (33.8–59.6)	35.1 (29.5–48.7)	41.9 (33.1–53.1)	ns
Xanthurenic acid (nmol/L)	11.8 (8.4–19.5)	9.2 (3.4–14.5)	19.9 (15–27.8)	0.0013
3-Hydroxyantralinic acid (nmol/L)	27.2 (22.5–35.5)	31.6 (20–47.7)	31.5 (23.4–41.1)	ns
Quinilonic acid (nmol/L)	349 (262.3–448.5)	474.4 (352.2–669.2)	359 (304–425)	0.039
K/T ratio	24.8 (21.2–30.8)	34.8 (31.2–46.9)	24.6 (20.8–28.9)	0.0001

^*^Kruskal-Wallis test was used for comparison between three groups, and Dunn’s Multiple Comparison Test for post-hoc pairwise analyses. Two-tailed Mann-Whitney U-test was applied for comparisons between two groups. NA (not available). ns (non significant) indicates p-value > 0.05.

**Figure 5:**
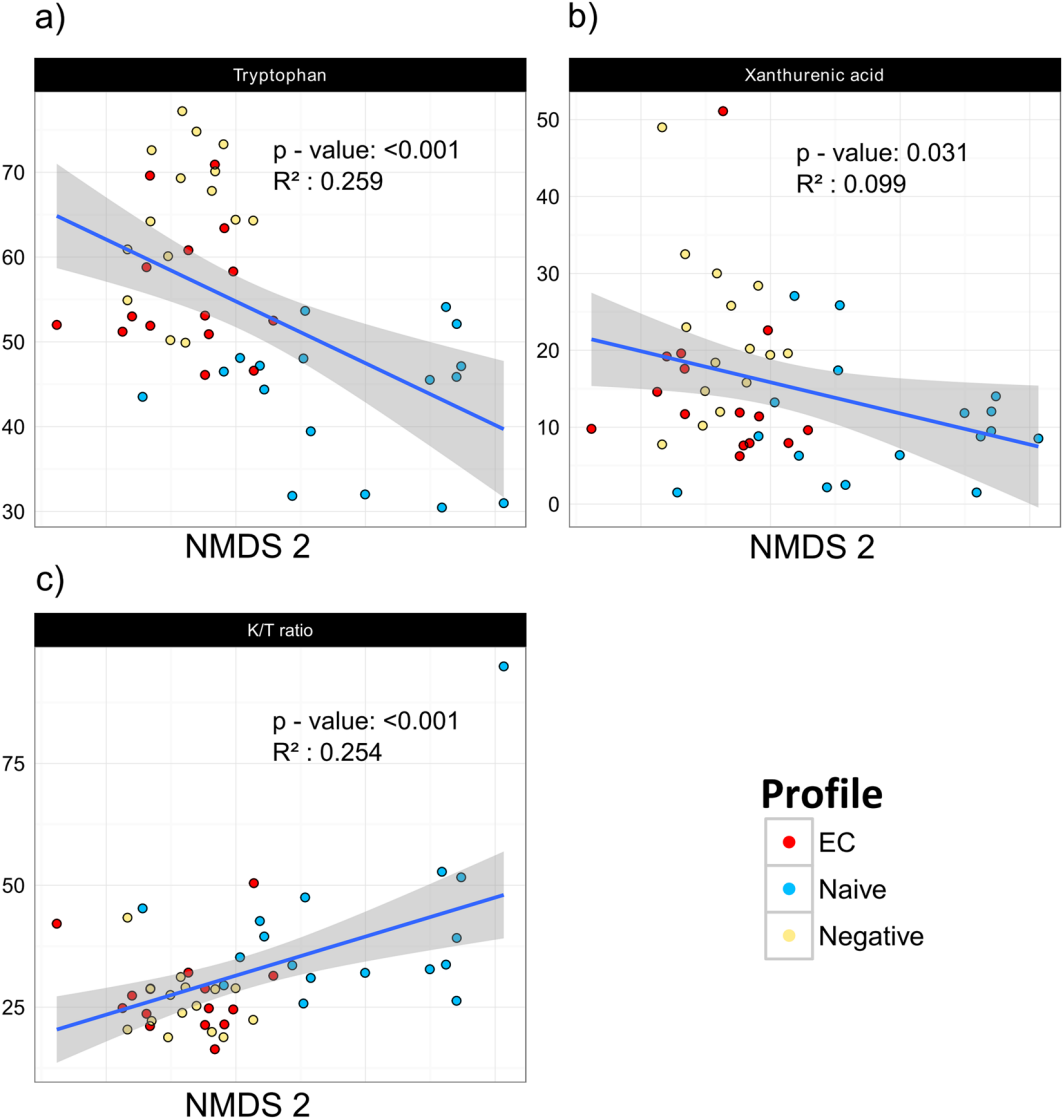
Correlations between tryptophan catabolism metabolites and NMDS 2 axis reveal clustering of naive patients. Significant correlations between NMDS 2 axis and tryptophan (**a**), xanthurenic acid (**b**) and K/T ratio (**c**) were observed, separating naive patients from EC and negative controls. The gray area defines the 95% confidence interval for the linear regression coefficients. The different groups are represented by different colors (EC-red, naive-blue, negative-yellow). Spearman’s correlation was applied for testing correlations between metabolites and NMDS plot axis coordinates.

Tryptophan levels in plasma were reduced in naive as compared to both EC and negative. Additionally, the naive group had several divergent levels of metabolites. Thus, xanthurenic and kynurenic acid levels were lower in naive as compared to negative; in contrary anthralinic acid levels and kynurenine/tryptophan (K/T)-ratio were increased in naive vs EC/negative. K/T-ratio was correlated to the number of observed genera (r = −0.47, p = 0.0009), richness indices: Chao-1 (r = −0.53, p = 0.0002) and ACE (r = −0.44, p = 0.002), but not to alpha-diversity indices. Significant correlations between levels of tryptophan, xanthurenic acid, K/T-ratio and NMDS2 axis were found (Table 3), mirroring a separation of naive from EC and negative in this axis (Fig. 5).

**Table 3:** Correlation strengths (R^2^) for each NMDS axis/marker.

	NMDS1	NMDS2
	R^2^	R^2^
hs-CRP (pg/ml)	−0.22	−0.06
LBP (ng/ml)	0.18	0.27
sCD14 (pg/ml)	−0.09	−0.29
Tryptophan (umol/L)	−0.20	−0.46*
Kynurenine (umol/L)	0.12	0.07
Anthralinic acid (nmol/L)	0.08	0.39
Kynurenic.acid (nmol/L)	−0.11	−0.18
3-Hydroxykynurenin (nmol/L)	0.05	−0.10
Xanthurenic acid (nmol/L)	−0.11	−0.29**
3-Hydroxyantralinic acid (nmol/L)	−0.08	0.02
Quinilonic acid (nmol/L)	0.08	0.28
K/T ratio	0.17	0.43*

*Indicates p-value < 0.01. **Indicates p-value < 0.05.

### Factors associated with the composition and functionality of gut microbiota

**Figure 6:**
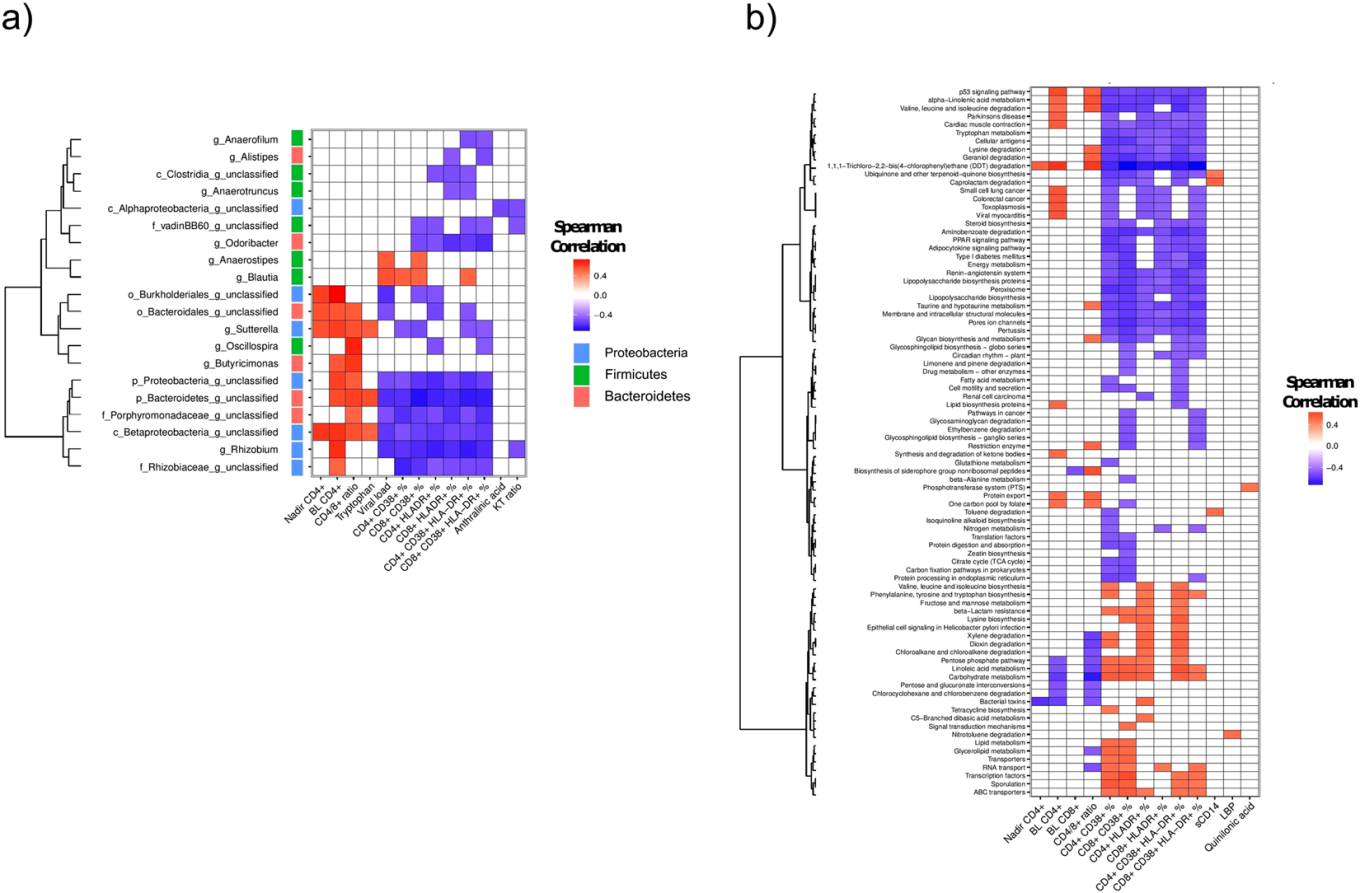
The composition and functionality of gut microbiota correlate with markers of immune activation and inflammation. Most cellular and some soluble markers of immune activation correlated to specific genera and functional pathways of gut microbiota. Correlations are presented by genus (**a**) and functional pathways (**b**). Spearman’s correlation was used. Associations with a Benjamini – Hochberg adjusted p-value lower than 0.01 were considered relevant. Immune activation and inflammatory parameters associated with less than two bacteria were discarded when plotting the heatmap.

We observed a distinct pattern of correlations between gut microbial composition, immunological markers and tryptophan catabolism (Fig. 6a). Interestingly, nadir and BL CD4+ T-cell counts, CD4/8+ T-cell ratio and tryptophan levels were strongly correlated to the abundance of genus *Sutterella*, whilst BL CD4+ correlated to *Rhizobium* and *Butyricimonas*. Moreover, CD4/8+ T-cell ratio was positively correlated to *Oscillopira* and *Butyricimonas*. Genera of *Sutterella*, *Oscillospira*, *Rhizobium*, *Anaerofilum*, *Alistipes*, *Anaerotruncus* and *Odirobacter* had all at least two inverse correlations with some of the cellular immune activation markers (CD38, HLA-DR). In contrary, abundance of *Blautia* was positively associated with immune activation (CD4+ CD38+, CD8+ CD38+, CD4+ CD38+ HLA-DR+ and VL). Additionally, unclassified genera of Burkholderiales, Bacteriodales, Proteobacteria, Betaproteobacteria and Rhizobiaceae were also positively correlated to BL CD4+ T-cell count. Inversely, there was a strong negative correlation between all of these taxa, unclassified genera of family Porphyromonadaceae, and most of the cellular immune activation markers. Only one of the identified genera, *Rhizobium*, was significantly inversely associated with K/T-ratio (Fig. 6a). There was an inverse correlation between BL CD4+ T-cell count, CD4/8+ T-cell ratio and several pathways related to carbohydrate metabolism, as also the essential omega-6 fatty acid linoleic acid. Conversely, alpha-linoleic acid (an essential n–3 fatty acid) metabolism was negatively associated to these markers. Furthermore, positive correlations were found between BL CD4+ T-cell count and synthesis and degradation of ketone bodies and lipid biosynthesis proteins pathways, both involved in lipid metabolism (Fig. 6b). CD4/8+ T-cell ratio was positively correlated to degradation of amino acids valine, leucine and isoleucine.

Cellular immune activation correlated with several pathways. Proportions of CD4/8+ (CD38+) T-cells were positively associated to carbohydrate metabolism, pentose-phosphate pathway (PPP) and also to overall metabolism of lipids and linoleic acid, whereas both fatty acid and alpha-linoleic acid metabolism were negatively correlated. Further inverse correlations were found between cellular immune activation and pathways involved in PPAR-signaling, steroid biosynthesis, adipocytokine signaling, citrate (TCA) cycle, degradation of amino acids, diabetes mellitus type I and tryptophan metabolism. Most of these associations were both significant for CD4+ and CD8+ (HLA-DR+/CD38+) T-cells. Only a few associations between soluble plasma markers sCD14 and LBP and microbiota function were found at the significance level of 0.01 (Fig. 6b), though additional correlations were observed at level 0.05 (Supplementary material Figure S3).

## Discussion

It has been widely accepted that HIV-infection is accompanied by immune activation, microbial translocation^2, 27–31^ and gut microbiota dysbiosis^8–10, 32^. Our study provides important observations concerning these pathogenic events in patients who spontaneously maintain sustained control of HIV, the elite controllers (EC). Thus, we present that their microbiota is richer and differs in predicted functionality from treatment naive HIV progressors, resembling the microbiota of HIV negative controls. We also confirm that the level of systemic immune activation and plasma markers of tryptophan catabolism pathway in EC are similar to uninfected individuals. Additionally we show that the microbiota richness is inversely correlated to K/T-ratio, a surrogate marker of IDO-1 activity, the rate limiting enzyme of systemic tryptophan catabolism.

To date, the mechanisms behind the viral control in EC are not fully understood. It has been postulated that more potent HIV-specific CD8+ T-cell responses, expression of restriction factors like APOBEC3 family proteins and enrichment of specific NK-cell receptors contribute to this persistent control of HIV^33^. Even if these individuals can suppress the virus, microbial translocation and chronic immune activation still feature the course of HIV-infection also in EC^7^.

The dysbiosis in progressive HIV infection has been described in several studies^30, 34, 35^. Albeit, even if only handful of EC has been included in these cohorts^8–10^, their microbiome diversity and composition have differed from HIV progressors. Our current study, which included the so far highest number of EC, confirms and expands the previous observations. We found that several ecological indices of EC microbiota (including richness and number of observed species) were significantly higher in EC as compared to naive and not different from matched negative controls. Additionally LASSO analysis showed a higher similarity between the microbiota of EC and negatives than that of viremic HIV infected individuals. Furthermore, we found that EC had a unique bacterial signature at genus level with 17 genera that were significantly differently distributed between the groups. Hence, *Succinivibrio*, *Sutterella*, *Rhizobium*, *Delftia*, *Anaerofilum* and *Oscillospira* were more abundant, whereas *Blautia* and *Anaerostipes* were depleted in EC.

In a previous work, initiation of ART was followed by higher abundance of *Succinivibrio* ^9^. Interestingly, the metabolic properties of Succinivibrionaceae family members have been associated with ART related immune recovery^36^. The study suggested that bacteria of this family have anti-inflammatory capacity by accumulating molecules involved in reduction of viral infections and inflammation.

Members of *Sutterella* genus are prevalent commensals in the GI-tract with mild pro-inflammatory capacity, except for *Sutterella wadsworthensis* whose pathogenic properties have been described recently^37^. The authors proposed that members of *Sutterella* may have different immunomodulatory roles, as *Sutterella* spp. except from *S. wadsworthensis* may elicit T_H_-17 differentiation by adhering to intestinal epithelial cells. Additionally, lower abundance of *Sutterella* has been found in the gut microbiome of patients with multiple sclerosis, and in Hodgkin lymphoma patients after allogenic hematopoetic stem cell transplantation^38, 39^. In our study, we present increased abundance of *Sutterella* in EC with several correlations to immune markers (positive with BL CD4+ T-cell counts and negative to markers of cellular activation). Thus, our findings warrant further characterization of *Sutterella* genus at species level to determine its involvement in the modulation of the immune system.

Similar to us, Mutlu *et al*. found decreased abundance of *Oscillospira* in HIV positive patients with progressive infection^32^. The strong positive correlation between the *Oscillosipira* and CD4/CD8 ratio suggests that this genus was associated with lower systemic inflammation in our cohort, which has also been shown in patients with Crohn disease and obesity^40^.

Conversely, depletion of *Blautia* and *Anaerostipes* has been described in patients with HIV infection^31, 32^, but instead we now report enrichment of *Blautia* and *Anaerostipes* in naive patients, linked to cellular immune activation. Additionally, we found increased abundance of genus *Rhizobium* in EC, with positive correlation to BL-CD4 counts and inverse to viral load, cellular immune activation markers and K/T-ratio. This bacterium, belonging to phylum Proteobacteria, has been attributed to nitrogen fixing properties in plants^41^. Interestingly, the K/T-ratio correlated only with genus *Rhizobium*, suggesting that this particular taxa may play a role in the bacterial metabolism of tryptophan. Moreover, similar to a previous study^42^, we found that the proportions of tryptophan metabolism related bacterial genes were depleted in naive as compared to both negative and EC. This probably reflects the loss of intraluminal commensal bacteria involved in tryptophan catabolism, like *Lactobacillus* spp^43^. Based on our results, we speculate that *Rhizobium* genus may be a factor orchestrating tryptophan degradation as *Rhizobium* members are able to convert tryptophan to indole-3-acetic acid^44^. The reduced ability of gut microbiota to produce tryptophan derived indole metabolites related to dysbiosis in progressive HIV-infection is known to affect the production of IL-22 by innate lymphoid cells which together with loss of T_H_-17 cells increase the disruption of the epithelial barrier and exacerbate overgrowth of pathogenic bacteria^43, 45, 46^. These events in the gut were mirrored by signs of increased microbial translocation and immune activation in naive group.

The metabolism of tryptophan along the kynurenine pathway in peripheral tissues (including skeletal muscle, liver and white blood cells) is mediated by several enzymes, but the main inducible and rate-limiting enzyme is Indolamine-2,3-Dioxygenase 1 (IDO-1)^47^. During HIV-infection, the IDO-1 activity is induced in dendritic cells by microbial products, and higher proportions of mucosal adherent bacteria possessing IDO homologs have been found in HIV-infected individuals^10^. Several tryptophan catabolites, e.g. 3-hydroxyanthranilic acid, influence T-cell activation and contribute to the loss of gut resident Th17+ T-cells and to alterations in the ratio between Th17 and regulatory T-cells^13^. Additionally, kynurenine has been found to impair the survival of memory CD4+ T-cells by inhibition of IL-2 signaling^48^. In concordance with results from other studies^10, 13, 49^, our data confirm that tryptophan metabolism in plasma is increased in progressive HIV-infection, but not in EC. Furthermore, IDO-1 activity (measured by K/T-ratio) correlated with NMDS2 axis, separating naive patients from the other groups, supporting that the gut microbiota composition affects systemic metabolism of tryptophan through kynurenine pathway. To our knowledge, we present the novel finding that IDO-1 activity is inversely correlated to the richness of gut microbiota, alike CD4/8 T-cell activation (data not shown). Up to now, most studies on probiotics supplementation have focused on suspension with single or very few bacterial species^50–54^. Our observation indicates that alternative therapeutic interventions modulating gut microbiota richness and not only composition are warranted in order to reduce HIV-related inflammation.

As illuminated by Moya and Ferrer^55^, not only the bacterial composition is important in a given microbiota. Other factors like stability, resistance, resilience, and redundancy contribute to the functional properties of the microbiome. During HIV-infection, shifts in gut microbiota have been associated with alterations of metabolites involved in epithelial barrier integrity, hepatic function, viral infectivity and inflammation, influencing the recovery and activation of T-lymphocytes.

Up till now, only a few studies included functional analysis of gut microbiota in HIV patients^10, 34, 36^. In our cohort, inferred functional analysis of microbiota revealed interesting changes of gene abundance between the groups. We found lower abundance of genes involved in metabolism of carbohydrates; instead lipid metabolism related genes were enriched in EC. These differences were observed at both KEGG levels. Obviously, intracellular metabolic pathways involved in carbohydrate and lipid metabolism (like glycolysis, PPP, oxidation and synthesis of fatty acids, amino acid metabolism) are major players regulating both innate and adaptive immune cells^56^. Given that the vast majority of immune cells are located in the gut, the availability of nutrients for the gut-resident immune cells and the local metabolic milieu may influence the immunometabolism in gut compartment, subsequently tuning the immunological architecture and response to microbial stimuli. For instance, the short-chain fatty acid butyrate, derived from commensal microbiota, has been found to preferentially induce differentiation of colonic regulatory T-cells by expression of *Foxp3* gene, mediated by butyrate driven epigenetic modifications promoting inhibition of histone deacetylases (HDACs)^57^. Also long chain omega 3- polyunsaturated fatty acids (PUFA), e.g. alpha-lineolic acid which in our study correlated positively to BL CD4+ T-cell count and negatively to immune activation, have immunomodulatory properties involved in activation, differentiation and signaling of CD4+ T-cells^58^. Additionally, improved gut microbiota composition and positive immunomodulatory effects have been associated with oral supplementation of the nutritional mixture including several prebiotic oligosaccharides and omega-3/6 fatty acids in ART naive HIV-infected subjects^59, 60^. Based upon these findings, we hypothesize that the composition and functional capacity of gut microbiota in EC may be one of the factors contributing to virological and immunological control of the HIV-infection in absence of ART. Even if the EC group was very similar to negative subjects at both compositional and inferred functionality analyses, there were still significant differences present between the Elite controllers and negative subjects.

We acknowledge the lack of extensive dietary data, which could bias our analysis. Additionally, gene functional profiles were inferred from 16S sequences. While inferred function has shown to be robust, particularly for gut microbiome^23^, they should be interpreted with caution. Our study was not designed to provide the answer about the association between the HIV progression and microbiota changes, which could be addressed in population studies with longitudinal design. On the other hand, our study was carefully designed regarding possible confounding and to our knowledge, we analyzed the microbiome of the largest cohort of EC described. Additionally, we cautiously report only correlations data which had a significance level <0.01, providing further strength to our results and conclusions.

In summary, we report that the microbiota of EC is different from individuals with progressive infection and more similar to HIV negative individuals. The differences are robust, present both in number of observed species, richness, composition and inferred functionality. Our data suggest the concept of microbiota related control of HIV infection in EC, presumably at metabolomics level. If confirmed by metabolomics studies, new intervention strategies to control HIV can be considered.

## Electronic supplementary material

Supplementary information
