# Supplementary material for: Richer gut microbiota with distinct metabolic profile in HIV infected Elite Controllers
Source: Sci Rep. 2017 Jul 24;7:6269. doi: 10.1038/s41598-017-06675-1 (PMC5524949; doi:10.1038/s41598-017-06675-1)
Supplement: Supplementary file 1 — Supplementary information [file 41598_2017_6675_MOESM1_ESM.pdf]

Supplementary information

Richer gut microbiota with distinct metabolic profile in HIV infected Elite Controllers

Jan VESTERBACKA[1,*], Javier RIVERA[2,*], Kajsa NOYAN[3], Mariona Parera[2], Ujjwal Neogi[3], Malu CALLE [4], Roger PAREDES[2,4,5,6], Anders SÖNNERBORG[1,3], Marc NOGUERA-JULIAN[2,4], Piotr NOWAK[1]

[1]Department of Medicine Huddinge, Unit of Infectious Diseases, Karolinska Institutet, Karolinska University Hospital, Stockholm, Sweden; [2]IrsiCaixa & AIDS Unit, Hospital Universitari Germans Trias i Pujol, Universitat Autònoma de Barcelona, Badalona, Spain; [3]Department of Laboratory Medicine, Division of Clinical Microbiology, Karolinska Institutet, Karolinska University Hospital, Stockholm, Sweden; [4]Universitat de Vic-Universitat Central de Catalunya, Catalonia, Spain; [5]Universitat Autònoma de Barcelona, 08193 Bellaterra, Catalonia, Spain; [6]HIV Unit & Lluita Contra la SIDA Foundation, Hospital Universitari Germans Trias i Pujol, Ctra de Canyet s/n, 08916 Badalona, Catalonia, Spain

*The authors contributed equally to this work

Supplementary Table 1. Metadata of study subjects

	EC (n=16)	Naive (n=32)	Negative (n=16)
**Antibiotics**[1]	2	6	2
**Probiotics**	1	2	1
**Special diet**[2]	2	0	1
**Smoking**	6	8	2
**Diabetes**	1	2	1
**Other medication**	11	9	4
**Travelled abroad**[3]	5	NA	1

[1]Within 3 months from inclusion

[2]Lactose, gluten free, vegan, vegetarian (EC: one lactose/gluten free diet, one vegetarian. Negative: one lactose/gluten free diet)

[3]More than 4 weeks during the last 12 months before inclusion

NA – not available

Figure S1

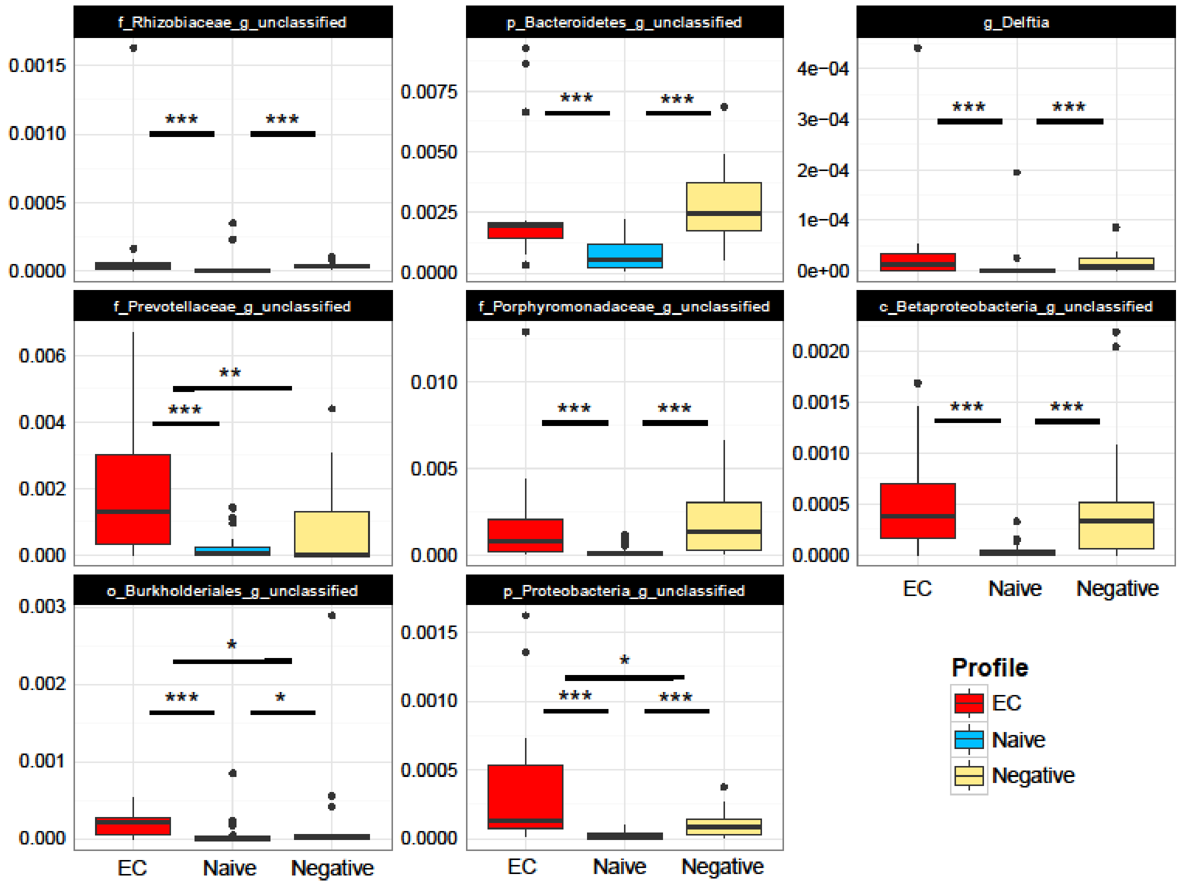

Figure S1. Significant differences in composition of fecal microbiota between EC, naive and negative. The abundance of bacterial taxa at genus level. Differences in taxa abundances were analyzed via Kruskal -Wallis ranked based test and Benjamini-Hochberg was used for correction of multiple testing. Adjusted p-value < 0.01 was considered significant for Kruskal-Wallis. Dunn`s post-hoc pairwise analyses: *p<0.05, **p<0.01, ***p<0.001. Box plots represent median (black horizontal line), 25th and 75th quartiles (edge of boxes), upper and lower extremes (whiskers). Outliers are represented by a single data point.

Figure S2

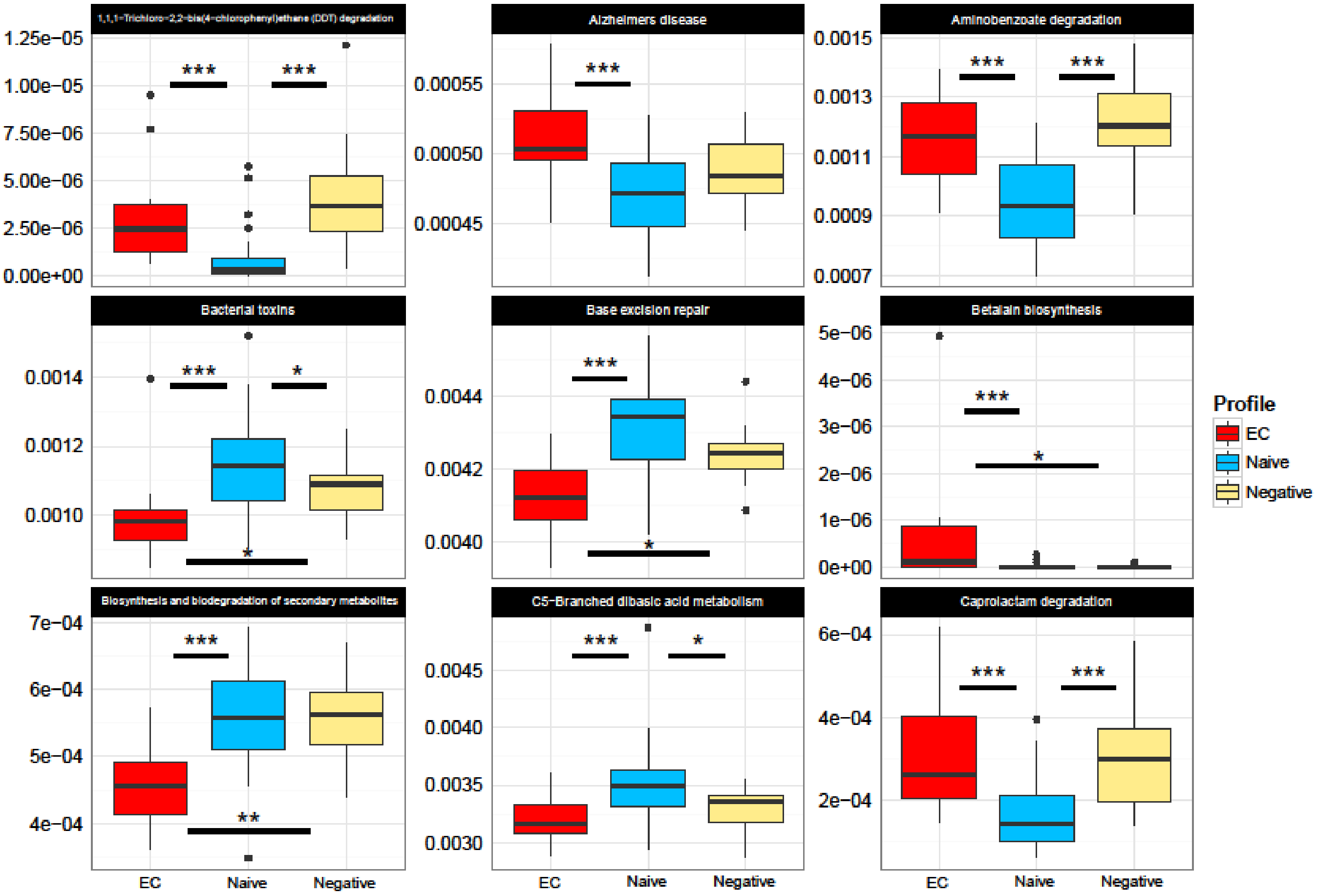

1,1,1-Trichloro-2,2-bis(4-chlorophenyl)ethane (DDT) degradation
1.25e−05
1.00e−05
7.50e−06
5.00e−06
2.50e−06
0.00e+00
***
***
Alzheimers disease
0.00055
0.00050
0.00045
***
Aminobenzoate degradation
0.0015
0.0013
0.0011
0.0009
0.0007
***
***
Bacterial toxins
0.0014
0.0012
0.0010
***
*
*
Base excision repair
0.0044
0.0042
0.0040
***
*
Betalain biosynthesis
5e−06
4e−06
3e−06
2e−06
1e−06
0e+00
***
*
Biosynthesis and biodegradation of secondary metabolites
7e−04
6e−04
5e−04
4e−04
***
**
C5−Branched dibasic acid metabolism
0.0045
0.0040
0.0035
0.0030
***
*
Caprolactam degradation
6e−04
4e−04
2e−04
***
***
EC
Naive
Negative
Profile
EC
Naive
Negative

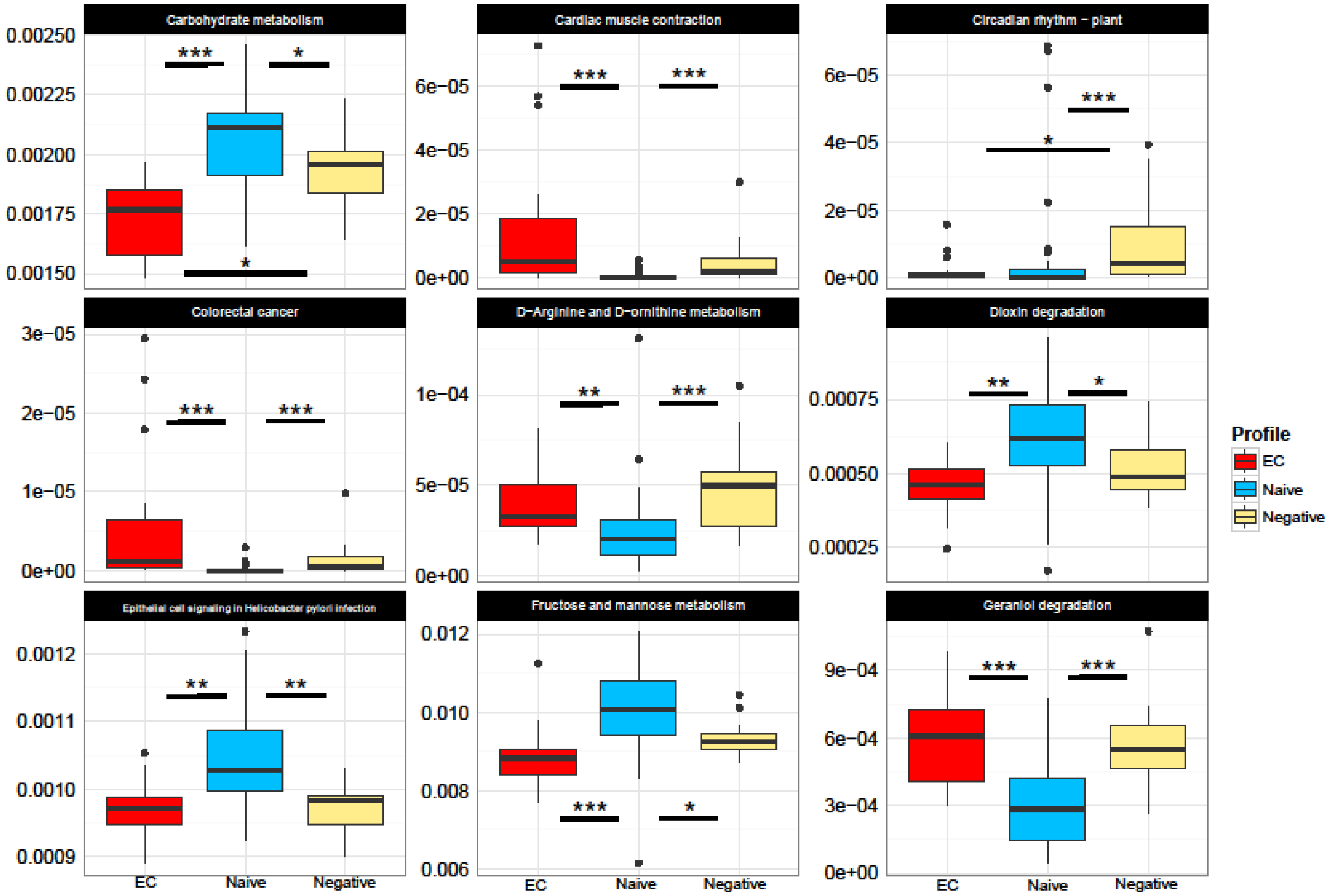

Carbohydrate metabolism
Cardiac muscle contraction
Circadian rhythm − plant
Colorectal cancer
D−Arginine and D−ornithine metabolism
Dioxin degradation
Epithelial cell signaling in Helicobacter pylori infection
Fructose and mannose metabolism
Geraniol degradation
Profile
EC
Naive
Negative

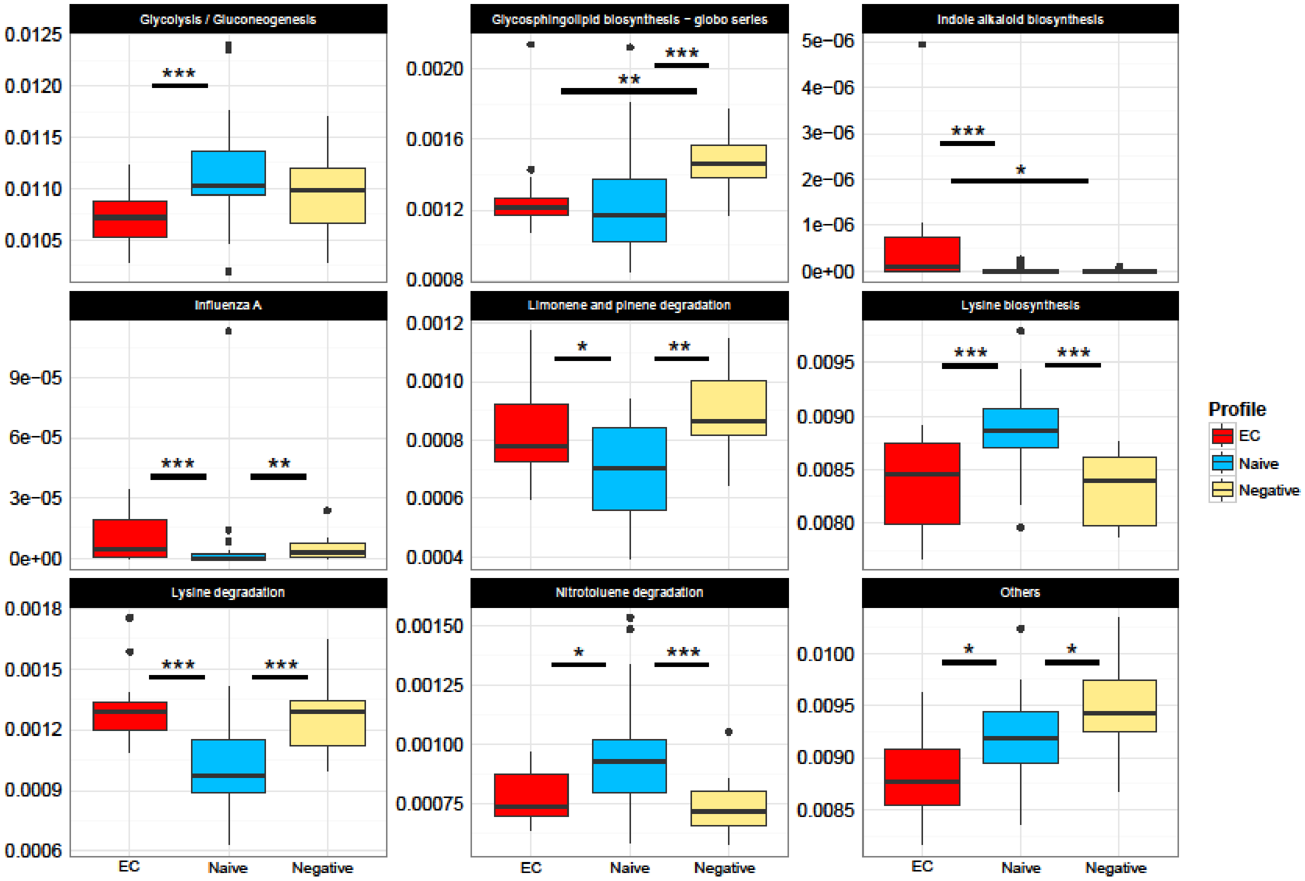

Glycolysis / Gluconeogenesis
Glycosphingolipid biosynthesis − globo series
Indole alkaloid biosynthesis
Influenza A
Limonene and pinene degradation
Lysine biosynthesis
Lysine degradation
Nitrotoluene degradation
Others
EC
Naive
Negative
Profile
EC
Naive
Negative

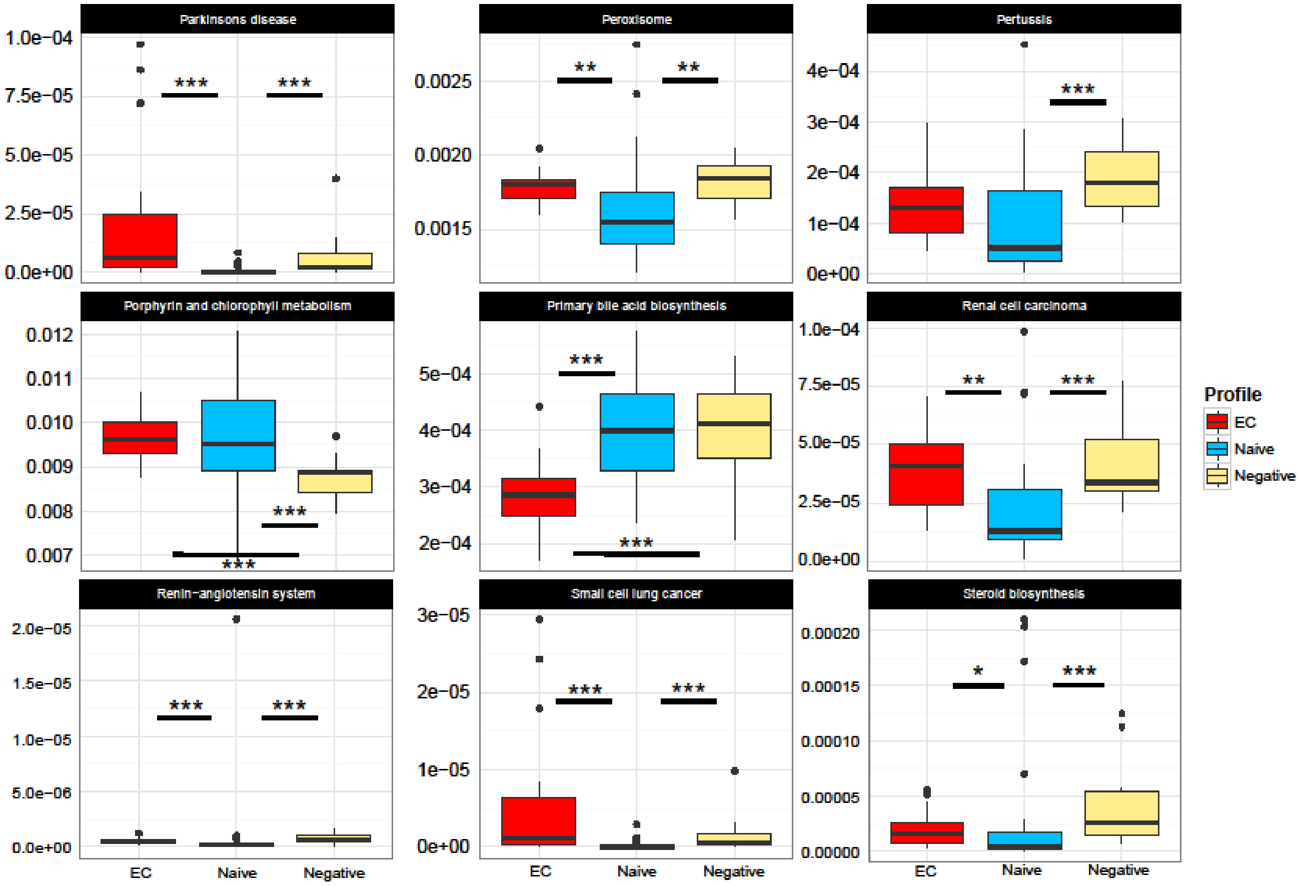

Parkinsons disease
Peroxisome
Pertussis
Porphyrin and chlorophyll metabolism
Primary bile acid biosynthesis
Renal cell carcinoma
Renin−angiotensin system
Small cell lung cancer
Steroid biosynthesis
Profile
EC
Naive
Negative

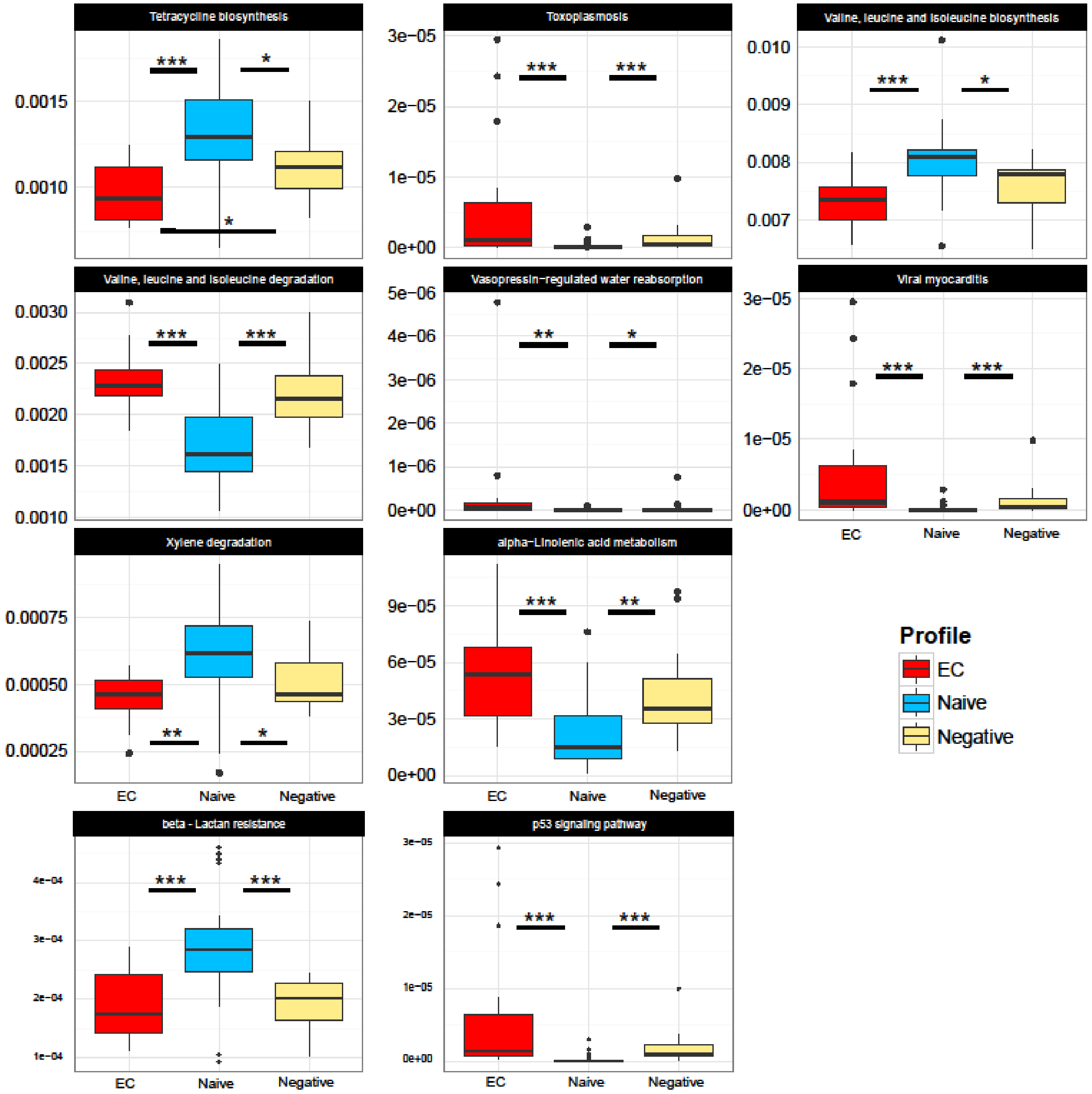

Figure S2. Predicted functional content of gut microbiota reveal significant differences between EC and naive/controls. Microbiome function was inferred using PICRUSt analysis. Differences in predicted gut microbiota functionality are shown in (a) and (b). Kruskal – Wallis rank-based test was applied, and Benjamini – Hochberg correction was used to correct for multiple testing. Adjusted p-value < 0.01 was considered significant for Kruskal-Wallis. Dunn`s post-hoc pairwaise analyses: *p<0.05, **p<0.01, ***p<0.001. Box plots represent median (black horizontal line), 25th and 75th quartiles (edge of boxes), upper and lower extremes (whiskers). Outliers are represented by a single data point.

Figure S3

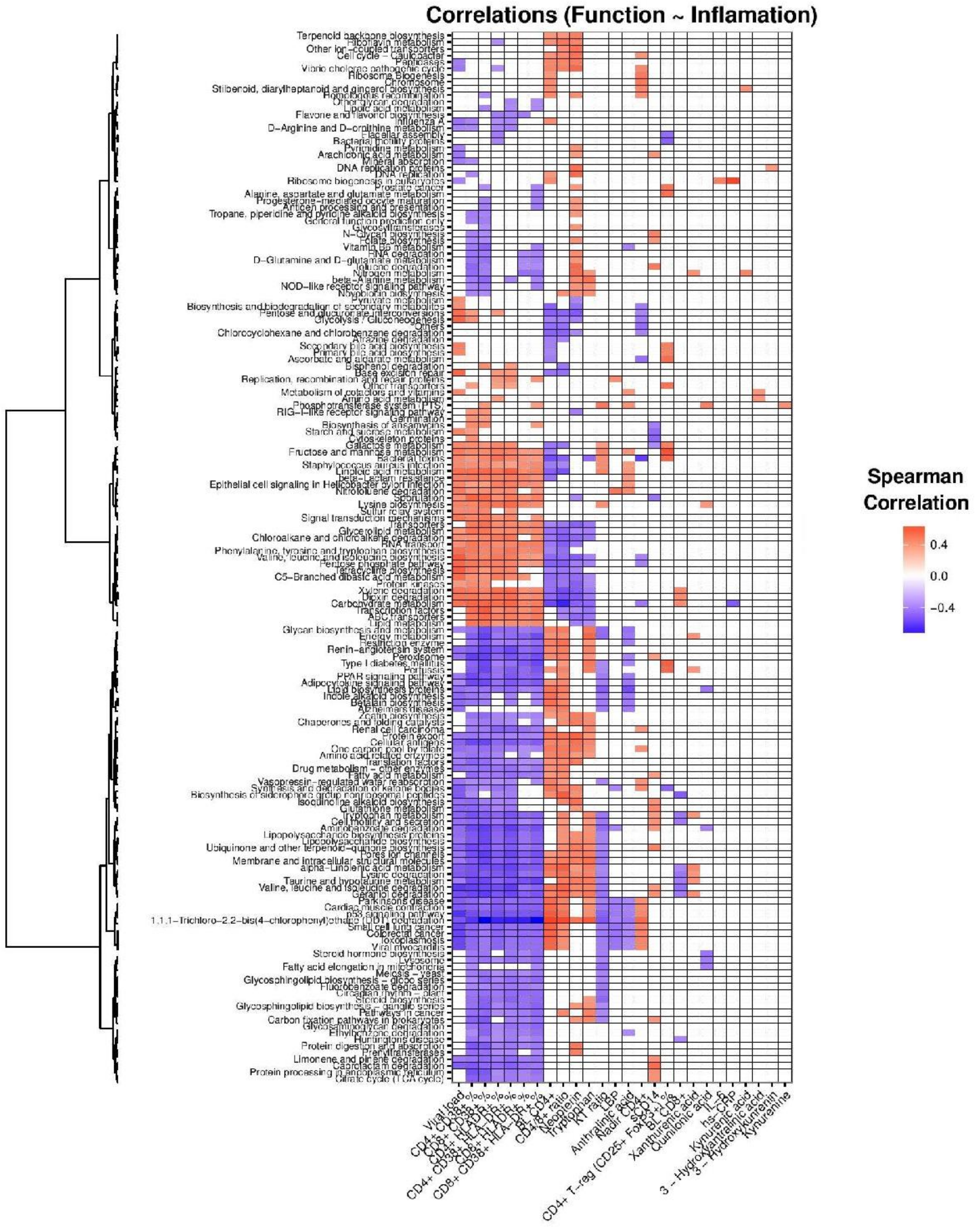

Figure S3. Significant correlations between predicted functional pathways and markers of immune activation and inflammation. All Spearman´s correlations with a Benjamini – Hochberg adjusted p-value lower than 0.05 are presented. Immune activation and inflammatory parameters associated with less than two bacteria were discarded when plotting the heatmap.
